# To update or to create? The influence of novelty and prior knowledge on memory networks

**DOI:** 10.1098/rstb.2023.0238

**Published:** 2024-07-29

**Authors:** Melanie J. Sekeres, Judith Schomaker, Lynn Nadel, Dorothy Tse

**Affiliations:** ^1^ School of Psychology, University of Ottawa, Ottawa, Ontario K1N 6N5, Canada; ^2^ Health, Medical & Neuropsychology, Leiden University, Leiden 2333 AK, The Netherlands; ^3^ Leiden Institute for Brain and Cognition, Leiden, The Netherlands; ^4^ Department of Psychology, University of Arizona, Tucson, AZ 85721, USA; ^5^ Department of Psychology, Edge Hill University, Ormskirk L39 4QP, UK

**Keywords:** schemas, memory network, novelty, hippocampus, medial prefrontal cortex

## Abstract

Schemas are foundational mental structures shaped by experience. They influence behaviour, guide the encoding of new memories and are shaped by associated information. The adaptability of memory schemas facilitates the integration of new information that aligns with existing knowledge structures. First, we discuss how novel information consistent with an existing schema can be swiftly assimilated when presented. This cognitive updating is facilitated by the interaction between the hippocampus and the prefrontal cortex. Second, when novel information is inconsistent with the schema, it likely engages the hippocampus to encode the information as part of an episodic memory trace. Third, novelty may enhance hippocampal dopamine through either the locus coeruleus or ventral tegmental area pathways, with the pathway involved potentially depending on the type of novelty encountered. We propose a gradient theory of schema and novelty to elucidate the neural processes by which schema updating or novel memory traces are formed. It is likely that experiences vary along a familiarity–novelty continuum, and the degree to which new experiences are increasingly novel will guide whether memory for a new experience either integrates into an existing schema or prompts the creation of a new cognitive framework.

This article is part of the theme issue ‘Long-term potentiation: 50 years on’.

## 1. Introduction

Imagine you are an academic who has recently transitioned to a new university. You are about to deliver your first research seminar during a colloquium series held by your new department. After entering the new lecture hall, you activate the projector screen and computer and survey the room before it fills with your colleagues. Although the environment and technology set-ups are novel to you, your experience in delivering research talks in similar lecture halls has provided you with prior knowledge, or a schema, for what to expect during the event.

Five minutes into your presentation, however, you are interrupted by a series of questions—this is very unlike the procedure at your former institution, where questions are held until the end. While this is initially a novel, unexpected experience, after attending the talks of others and giving several additional talks at your new institution, you will get accustomed to it. Delivering a talk in this new environment has updated your mental schema for conducting university seminars, although you retain a vivid memory of that first seminar experience at your new institution.

## 2. What is a schema?

The concept of the ‘schema’ is an enduring psychological idea that describes the process by which we develop scaffolded knowledge based on extractions from repeated experiences [1–3]. This scaffolding aids in integrating, understanding and remembering new related information—a process well-illustrated by the ‘research talk’ schema that enables academics to anticipate the flow of events during a presentation.

While the psychological underpinnings of schemas have been long established, it is only in recent years that the neurobiological mechanisms and systems underlying memory consolidation processes involved in schema formation have been elucidated. Recent neuroscience research posits that schemas consist of three fundamental components: first, they form an adaptable framework that incorporates associative knowledge from repeated past experiences; second, this framework facilitates and accelerates the processing of new information, encompassing encoding, consolidation and retrieval, thereby shaping future behaviour; and third, they are underpinned by a complex network of interconnected neocortical representations, with the hippocampus/medial temporal lobe (MTL) and medial prefrontal cortex (mPFC) playing pivotal roles in their formation and function [4–10]. Accumulating research from both rodent and human studies has begun to unravel the roles of the hippocampus and mPFC within the schema memory network, and their contributions to episodic and schematic memory processing may differ depending on the degree to which novel experiences match pre-existing schematic representations [4,5,11–15].

## 3. How do unique experiences become schematic?

At the systems neuroscience level in humans, several models have been proposed to account for how schemas develop from episodic memories [6,9,11,16,17]. The initial process of episodic memory consolidation transpires at the cellular/synaptic level, which is followed by a prolonged, dynamic process of systems consolidation and memory transformation throughout the recollection network, notably within the hippocampus and mPFC [18,19]. Shortly after novel events are first experienced and encoded, the hippocampus plays a critical role in the retrieval of the episodically detailed elements of the experience. Over time and with repeated reactivations of the memory, distributed representations form within the hippocampus and mPFC [20].

The hippocampus may continue to support the unique episodic event memory, with fine-grained details (colours, sounds, locations, context details and actions) represented within the posterior hippocampus. The perceptually detailed and event-specific elements characterizing the experience can become less relevant, and the coarsely detailed elements of the event being supported by the anterior region of the hippocampus may be sufficient to recall the gist of the event [21,22]. The highly detailed, event-specific elements of the memory may continue to be supported by the posterior hippocampus but may remain latent unless sufficiently cued at retrieval [23,24]. With repeated similar experiences (e.g. giving a research talk at different symposia), the statistical regularities of the event will be extracted to form an event-general, schematic representation or ‘framework’ for the event (e.g. research talk schema) that is reliant on ventromedial PFC (vmPFC) [9,20]. Connections between the anterior hippocampus and vmPFC support the interaction between gist elements of the event and the assimilation of common elements from past related experiences into the vmPFC-mediated schema network [20,25–27]. The development of the schematic representation or framework for the event does not replace the episodically detailed representation of each unique event experience within the hippocampus but rather can serve to guide the memory construction and elaboration processes as these representations interact dynamically during encoding and retrieval [28,29]. In this case, an activated schematic framework helps in rapidly understanding and guiding the encoding of new related experiences, while the new related experiences also contribute to the ongoing development and adaptation of the schematic representation for a class of events [28,29].

Evidence from animal models provides insight into the neural mechanisms underlying the episodic schema consolidation process. The traditional view of systems memory consolidation is that it is a gradual process that takes place over days or weeks (in rodents) or months and years (in humans) [30]. Within this approach, the hippocampus is thought to be involved in the rapid encoding of specific events, while the neocortex is thought to be involved in slower learning and generalization [31,32]. While the mPFC comes to support remote and schematic memories, its involvement begins early in the consolidation process [33], and these early interactions with the hippocampus appear to be critical for the time-dependent dendritic growth and spine remodelling in the mPFC [34,35]. In line with these insights, animal studies involving olfactory conditioning in rats reveal that the enhanced acquisition of odour memories is accompanied by long-term modifications in neuronal properties [36,37]. These findings underscore the complex interplay between immediate encoding mechanisms and the gradual integration of experiences into schemas.

If an appropriate schema has been previously established, new information can be rapidly incorporated via expedited systems consolidation [5,8]. Using a hippocampal-dependent paradigm, rats were trained to learn a schema involving six flavour–place paired associates (PAs) [4,5,38,39]. Following schema acquisition, rats were exposed to new PAs, followed by hippocampal lesioning at different timepoints to identify the necessity of the hippocampus in memory updating of an established schema [4]. When hippocampal lesions were made 3 h after new learning, memory was still reliant on the hippocampus. However, when lesions were made 48 hours after new learning, the hippocampus was no longer necessary for successful memory retrieval of the new PA location. Once the consistent schema was established, relevant new information became assimilated into cortical areas and rapidly became hippocampal-independent. In recent studies, Tse *et al*. and Takeuchi *et al*. [5,40] have utilized immediate early genes as neuronal markers to explore the neural underpinnings of memory encoding and consolidation. They highlight a robust association between midline neocortical (including the prelimbic cortex (PrL), anterior cingulate cortex (ACC) and anterior retrosplenial cortex (aRC)) and hippocampal connectivity. This neural connectivity is crucial for the effective encoding of new PAs, particularly when these associations fit within an existing, consistent schema. At the neural level, α-amino-3-hydroxy-5-methyl-4-isoxazolepropionic acid (AMPA)-mediated activity and *N*-methyl-d-aspartate receptor (NMDA)-mediated glutamatergic activity in mPFC (PrL, ACC) are necessary for assimilating new paired associates into an existing schema [5,39]. These findings support the existence of a coordinated functional network between the hippocampal formation and neocortical regions, which is essential for encoding new information in the context of the existing knowledge. Furthermore, these animal studies indicate that the systems consolidation of newly integrated information is expedited.

These findings provide a useful framework for understanding how unique episodic events contribute to the development of a schema for related experiences and how the brain consolidates and updates these memory representations with time and experience. The component of adaptability is essential to a flexible schema that may be updated based on schema-congruent new experiences. A key focus of this review is to explore when adapting an existing schema becomes counterproductive. This occurs when the disparity between a new experience and prior knowledge is so significant that, instead of assimilating information into an existing schema, creating a different representation becomes necessary.

## 4. When an experience does not quite fit the schema

van Kesteren *et al*.’s SLIMM model (schema-linked interactions between medial prefrontal and medial temporal regions) proposes how both schema-consistent and novel, schema-inconsistent memories are facilitated by different neural mechanisms, dependent on the mPFC and MTL, respectively [11]. This network detects congruency or ‘resonance’ between new information and an existing schema, where newly encountered information that resonates with prior knowledge will activate relevant schema representations in the mPFC, which in turn inhibits the MTL and expedites consolidation for these associations within the neocortex. Newly encountered events or stimuli that are incongruent or that have low resonance with pre-existing schema networks will produce a high degree of prediction error, which will likely engage the hippocampus to encode the novel information as part of an episodic memory trace [11,41], potentially leading to strong recollection of the novel information [42].

Novel experiences or events that do not resonate with the existing schema—and are thus schema-incongruent—may be highly memorable owing to their continued engagement of the hippocampus over time. Bonasia *et al*. [14] found that encoding and retrieving film clip events judged to be congruent with commonly experienced everyday events (e.g. a family having dinner) strongly engaged the vmPFC and elicited high functional connectivity between mPFC and cortical regions associated with semantic processing, whereas encoding and retrieval of highly novel or schema-incongruent events (e.g. a woman squeezing cheese) more strongly engaged MTL (hippocampus, parahippocampus) regions. When assessing the accuracy of these memories one week after encoding, schema-congruent clips were recalled with numerous errors. This degraded retrieval for more congruent information suggests a default to a stereotypical, distorted or generalized and decontextualized version of the event, as the memory for the film clip event loses specific details during the schema assimilation process [6,21,43,44]. Memory accuracy for the schema-incongruent clips, however, remained consistent over seven days following encoding, suggesting a high degree of fidelity for novel event memories, supported by continued MTL engagement. These findings are in line with the ‘novelty-encoding hypothesis’ that memory is typically enhanced for novel rather than familiar information [45,46] and consistent with predictions of the SLIMM model [11]. These data support the notion that schema congruency and novelty differentially mediate vmPFC and MTL activity to facilitate memory encoding and retrieval, with the latter promoting formation of a new, perceptually detailed episodic memory trace.

## 5. How does the brain ‘decide’ whether to update the schema or to create a new memory trace?

It has been suggested that the hippocampus identifies different types of novelty and communicates this to the substantia nigra (SN) and ventral tegmental area (VTA) via the striatum [47–49], resulting in dopamine (DA) release in the hippocampus. In the SN/VTA, these signals influence both phasic and tonic DA activities. Düzel *et al*. [47] proposed that phasic DA triggers the creation of plasticity-related products, while tonic DA activity raises the likelihood of phasic bursts, thereby facilitating learning. Contrary to the belief that these effects are solely mediated by tyrosine hydroxylase-expressing (TH+) neurons in the VTA, recent animal findings highlight the locus coeruleus (LC) as a critical player [50,51]. LC neurons are highly responsive to environmental novelty and possess a denser projection to the hippocampus compared with VTA neurons. Furthermore, optogenetic stimulation of LC TH+ neurons induced memory enhancements akin to those observed following novel events, and these effects persisted even with VTA inactivation. Novelty can thus increase hippocampal DA via both the LC and VTA pathways. Whether the effects of novelty are mediated by the LC or VTA pathway, however, may depend on the type of novelty.

**Figure 1.**
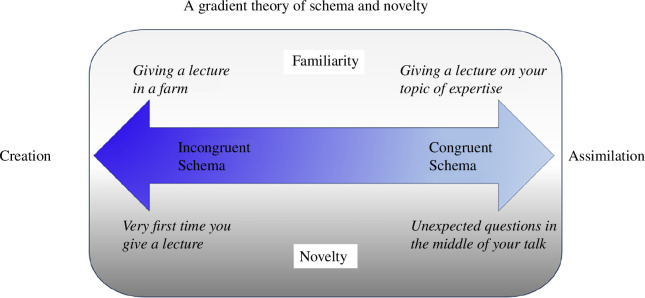
A gradient theory of schema and novelty. Events can be schema congruent and familiar (e.g. giving a lecture on your topic of expertise). Events may also be familiar by themselves but may occur in an incongruent context (e.g. giving a lecture on a farm). Although we may have very specific expectations with regard to familiar events, such prior knowledge typically lacks for novel events. To some extent, even novel events may be schema-congruent (e.g. getting unexpected questions during the middle of your talk) but can also be schema-incongruent (e.g. the very first time you give a lecture). Congruent information leads to assimilation in an existing schema. In contrast, incongruent information—either novel or familiar—may lead to updating of memories and in extreme cases may result in the creation of a new schema network.

Duszkiewicz *et al*. [52] proposed that novel experiences can be put on a spectrum ranging from somewhat different from a previous experience but with overlap to the previous experience, to evidently novel experiences that are different from all previous experiences. Although they suggest that novel events occur on a continuum, they propose that two separate neural mechanisms underlie the mnemonic processes at the extreme ends of this spectrum. In their framework, Duszkiewicz *et al*. [52] termed the type of novel events that have overlap with previous experiences ‘common novelty’ and identified the VTA as the source of hippocampal DA for this type of novelty. This type of information resembles previous experiences and as such could be easily assimilated into existing memory schemas. In contrast, they propose that ‘distinct novelty’—novel events that share minimal overlap with previous experiences—activates the LC with potentially different outcomes. Neuroimaging studies in humans have revealed that both the LC and VTA are activated by different types of novelty (e.g. stimulus novelty activating the VTA [53] and congruent novelty and deviant novel oddball stimuli activating the LC [54,55]; for a discussion of these different types of novelty in the literature in humans, see below). Although the neural mechanisms underlying these two types of novelty (i.e. common and distinct novelty) as identified by Duszkiewicz *et al*. [52] on this spectrum are proposed to be unique, our experiences may rather lie on a spectrum of extreme familiarity to extreme novelty (see figure 1).

Recent advances in genetic and optogenetic techniques have enabled more precise investigations into the roles of neuromodulation, particularly by the LC and VTA, in reacting to novelty and influencing learning and memory. Studies leveraging these methods have uncovered that DA release from LC projections plays a crucial role in learning and memory tasks [50,51,56,57], with optogenetic stimulation enhancing both DA and norepinephrine levels in the hippocampus [56]. This has established a direct causal link between LC-derived DA release and improved performance in spatial and novelty-associated memory tasks. Notably, blocking noradrenergic (NA) activity did not impact these memory enhancements [50,56], a finding that contrasts with previous research emphasizing the importance of beta-adrenergic signalling in memory encoding [58,59]. These findings highlight the co-release of DA and NA from the LC, and the significant contribution of LC-derived DA to memory processes. However, the distinct roles of DA and NA from the LC and VTA in memory processes remain to be fully elucidated [60].

In the human literature, a similar distinction has been made. Related to the term distinct novelty, the term ‘stimulus novelty’ is often used to refer to novel events that bear minimal resemblance to earlier experiences, while ‘contextual novelty’ refers to something being new within a specific context or environment, without being novel in and of itself [61]. Electrophysiological studies have shown that (contextually) novel stimuli evoke typical event-related potentials, such as the N2 (approx. 200–350 ms post-stimulus) and slightly later in time the P3 (approx. 350–550 ms post-stimulus), with the earlier negative event-related potential components associated with the detection of novelty/deviance, and the later positive-going component associated with orienting towards or evaluating novelty [62–64]. Interestingly, the processing of novelty not only depends on the novelty of the stimulus itself but is also affected by the context in which it occurs. Schomaker *et al*. [65] found that the novelty P3 was diminished when novel stimuli occurred in a complex stimulus environment or when novel stimuli were frequent. This suggests that the way novelty is processed depends on whether it is expected (congruent, e.g. giving a seminar on your topic of expertise) or unexpected (incongruent, e.g. unexpected questions during your talk) in that context (see figure 1). Interestingly, these electrophysiological novelty responses—such as the event-related P3 component in humans—have been linked to the noradrenergic LC system [66,67]. Work in animals has suggested that noradrenergic LC activity provides a network reset that allows adaptation to a rapidly changing environment [68,69] by the reorientation of attention and by supporting cognitive flexibility [70]. LC neurons respond to novelty or other salient stimuli, and the associated release of NA could promote sensory processing and retrieval of semantic contexts from memory. The amygdala responds to novelty as well [71,72] and amygdala activity during encoding has been linked to later memory success [73]. One study in younger and older human adults found a functional link between the amygdala and hippocampal region CA3 specifically for memory for semantically congruent events [74], suggesting that the amygdala may play a modulating role in schema-dependent memory. Through its interconnections with the hippocampus, the amygdala is also activated in response to the prediction error generated by schema-incongruent information in the hippocampus and the need to update the emotional value of the new stimuli or information that is inconsistent with prior knowledge or associations [29,75,76]. In case of a mismatch, a network reset could support the distinct encoding of novel events and could contribute to the formation of novel networks via effects on long-term potentiation. The question remains: when is information assimilated to an existing schema, and when, instead, is it the basis for forming an entirely new memory?

Here, we propose a gradient theory of both schemas and novelty, as illustrated in figure 1. Events can range from being congruent and familiar, such as repeatedly delivering the same lecture for years, to being familiar yet presented in an incongruent context, such as lecturing at a new university. While familiar events usually come with set expectations, novel events often lack such prior knowledge. Even novel events might align with the existing schemas to some degree (for instance, receiving unexpected questions during a lecture), or they might be completely schema-incongruent (such as giving a lecture for the first time). Congruent information that aligns with the existing schemas is typically *assimilated* into those schemas, a process facilitated by the hippocampus and the vmPFC. Conversely, incongruent information that does not align—whether novel or familiar—might lead to the updating of memories or, in more extreme cases, to the creation of new schema networks, potentially involving the hippocampus and the LC.

When new information is encountered, the hippocampus may act as a pointer, or index, to compare the new information with the existing schemas stored in various cortical regions [17,77,78]. If the new information closely aligns with an existing schema, the brain integrates this information into that schema. Tse *et al*. [4] conducted a study where rats were trained with both consistent and inconsistent schemas. Their findings showed that when a consistent schema is present, rats can quickly learn new PAs in just one trial. However, in the absence of an established schema, the rats faced challenges in rapidly learning new PAs. Following that, Alonso *et al*. [7] suggested that the involvement of the hippocampus and the cortex varies as a function of the extent and quality of prior knowledge. As one progresses from being a novice to being an expert, the hippocampus’ essential role in encoding finely detailed elements of each experience diminishes, and cortical regions become more capable of supporting the schematic representation of the class of congruent experiences. The degree to which subsequent related experiences are congruent with the schematic representation will determine the role of the anterior and posterior hippocampus, with the latter’s role diminishing with further schema-congruent experiences. Based on their model, when new information significantly differs from the existing schemas, creating a notable discrepancy, updating the current schema would be inefficient or inadequate. The prediction is that hippocampal activity should increase during the encoding and consolidation of novel experiences and associations because these processes contribute to the formation of a new memory trace and its related pairings. With time and with repeated similar experiences, these novel traces could eventually develop their own schema representation, at which point the mPFC would again become increasingly engaged [11].

We rarely experience stimuli and events that are completely unfamiliar, and new experiences instead reflect some combination of new and old stimuli/events. Given this, a new experience will activate an existing schematic representation within the vmPFC that incorporates the familiar elements. This activated schema will then trigger the hippocampus to evaluate newly encountered event details within the context of the activated schema. If these event details are sufficiently congruent with the existing schema, the hippocampus encodes just the new elements of the event, which become rapidly assimilated into the schema [21]. If the event details are sufficiently novel and totally incongruent with the activated schema, the hippocampus will instead form a new representation of the event [9], encoding its fine-grained perceptual elements[76] within the posterior hippocampus as well as forming gist-like representations of the event in anterior hippocampus. As we learn more about the dynamics underlying these hippocampal processes, we will come closer to understanding how novel experience interacts with prior knowledge to optimize new learning and adaptive behaviour.

## 6. Conclusion

Reflecting on the historical progression since the concept of schemas was first introduced in the 1930s [1], it is clear that our understanding of their neurobiological underpinnings has expanded significantly, especially with recent advancements in neuroscience. However, this expansion of knowledge also brings to light new questions, particularly concerning the exact processes through which the existing cortical knowledge is updated. Our proposition that the fate of memories is influenced by both the novelty of the information and its congruence with existing schemas opens new avenues for exploration. Notably, while the neurobiological mechanisms at the extremes of this spectrum are relatively well-understood, those governing the formation and integration of memories in the intermediate range remain less clear. Understanding the mechanisms of memory formation and schema adaptation is essential for a deeper comprehension of human cognition and behaviour and could have significant implications for addressing various psychological and neurological disorders.

## Data Availability

This article has no additional data.
